# Molecular dynamics simulations in photosynthesis

**DOI:** 10.1007/s11120-020-00741-y

**Published:** 2020-04-15

**Authors:** Nicoletta Liguori, Roberta Croce, Siewert J. Marrink, Sebastian Thallmair

**Affiliations:** 1 Department of Physics and Astronomy and Institute for Lasers, Life and Biophotonics, Faculty of Sciences, De Boelelaan 1081, 1081 HV Amsterdam, The Netherlands; 2 Groningen Biomolecular Sciences and Biotechnology Institute & Zernike Institute for Advanced Materials, University of Groningen, Nijenborgh 7, 9747 AG Groningen, The Netherlands

**Keywords:** Molecular dynamics, Photosynthesis, Light harvesting, Thylakoid membrane, Conformational switch, Coarse-grained

## Abstract

Photosynthesis is regulated by a dynamic interplay between proteins, enzymes, pigments, lipids, and cofactors that takes place on a large spatio-temporal scale. Molecular dynamics (MD) simulations provide a powerful toolkit to investigate dynamical processes in (bio)molecular ensembles from the (sub)picosecond to the (sub)millisecond regime and from the Å to hundreds of nm length scale. Therefore, MD is well suited to address a variety of questions arising in the field of photosynthesis research. In this review, we provide an introduction to the basic concepts of MD simulations, at atomistic and coarse-grained level of resolution. Furthermore, we discuss applications of MD simulations to model photosynthetic systems of different sizes and complexity and their connection to experimental observables. Finally, we provide a brief glance on which methods provide opportunities to capture phenomena beyond the applicability of classical MD.

## Introduction: towards a dynamic structural view of photosynthesis

The photosynthetic membrane, or thylakoid, is a continuous membrane system consisting of a lipid bilayer composed mainly of galactolipids and phospholipids (Duchêne and Siegenthaler 2000), with embedded protein complexes and cofactors. The thylakoid separates the aqueous phase of the chloroplast into different domains: the inner portion is called the lumen while the outer one is known as the stroma. In this membrane, the initial steps of photosynthesis, collectively known as the light reactions, take place. Via these reactions, electrons are removed from water and transported across the membrane while protons are pumped into the lumen, creating a proton gradient across the membrane. Electrons and the proton gradient are used to provide adenosine triphosphate (ATP) and nicotinamide adenine dinucleotide phosphate (NADPH) to the downstream, “dark” reactions of the Calvin–Benson–Bassham cycle, which produce carbohydrates from water and CO_2_ (Blankenship 2014; Croce et al. 2018).

The light reactions are regulated principally by four different integral membrane multi-protein complexes binding pigments and other cofactors. These complexes can be classified as follows: two photosystems, PSII and PSI which are large pigment-binding protein complexes [ ≫ 500 kDa (Heinemeyer et al. 2004)] involved in light-harvesting and light-driven electron transfer processes, cytochrome (Cyt) b_6_f, a > 200 kDa complex also involved in electron transfer (Baniulis et al. 2008), and the ATP-synthase, which is a ≫ 500 kDa enzyme active in proton-driven synthesis processes (Seelert et al. 2003). Additional players involved in regulatory mechanisms are also present in the thylakoid (Rochaix 2014).

In oxygenic photosynthetic organisms, visible and near-infrared sunlight energy is mainly absorbed by chlorophylls (Chls). Chls are tetrapyrroles and can be found in nature with different substitutions on their pyrrole ring. Some of such substitutions significantly tune the absorption spectra of the Chls (Scheer 1991; Kühl et al. 2005; Chen et al. 2010; Büchel 2019). Different photosynthetic organisms adopt distinct types of Chls with absorption properties matching the light spectrum available in their natural habitat (Croce and van Amerongen 2014; Stomp et al. 2007). In addition to Chls, photosynthetic organisms use carotenoids (Cars) and phycobilins to increase their absorption cross section in the green region (500–600 nm) (Beale 1993; Frank and Cogdell 1996), which is poorly absorbed by Chls.

Photosynthetic pigments are bound to the proteins that constitute the cores of PSII and PSI and to the outer antennae: the light-harvesting complexes (LHCs) or the phycobilisomes (Croce and van Amerongen 2014). The light-harvesting complexes are functionally connected to the cores of PSII and PSI forming “supercomplexes” (Gao et al. 2018). For a detailed review on the light-harvesting building blocks of photosynthetic organisms we refer the reader to Croce et al. (2018). The role of an antenna is to absorb solar photons and transfer the excitation energy to the cores where charge separation occurs. The pigment-to-protein ratio is generally very high in the antennae: in the case of plants and green algae, for example, ~ 25 kDa of protein can bind ~ 15 kDa of pigments (Nicol and Croce 2018). Such a large pigment/protein ratio results in crowded supercomplexes, e.g., in the example of the largest PSII supercomplex isolated from plants (Caffarri et al. 2009), this packing corresponds to a dimeric core binding 18 LHCs for a total of ~ 314 Chls and ~ 88 Cars and 4 pheophytins plus additional cofactors and lipids (Su et al. 2017).

**Fig. 1:**
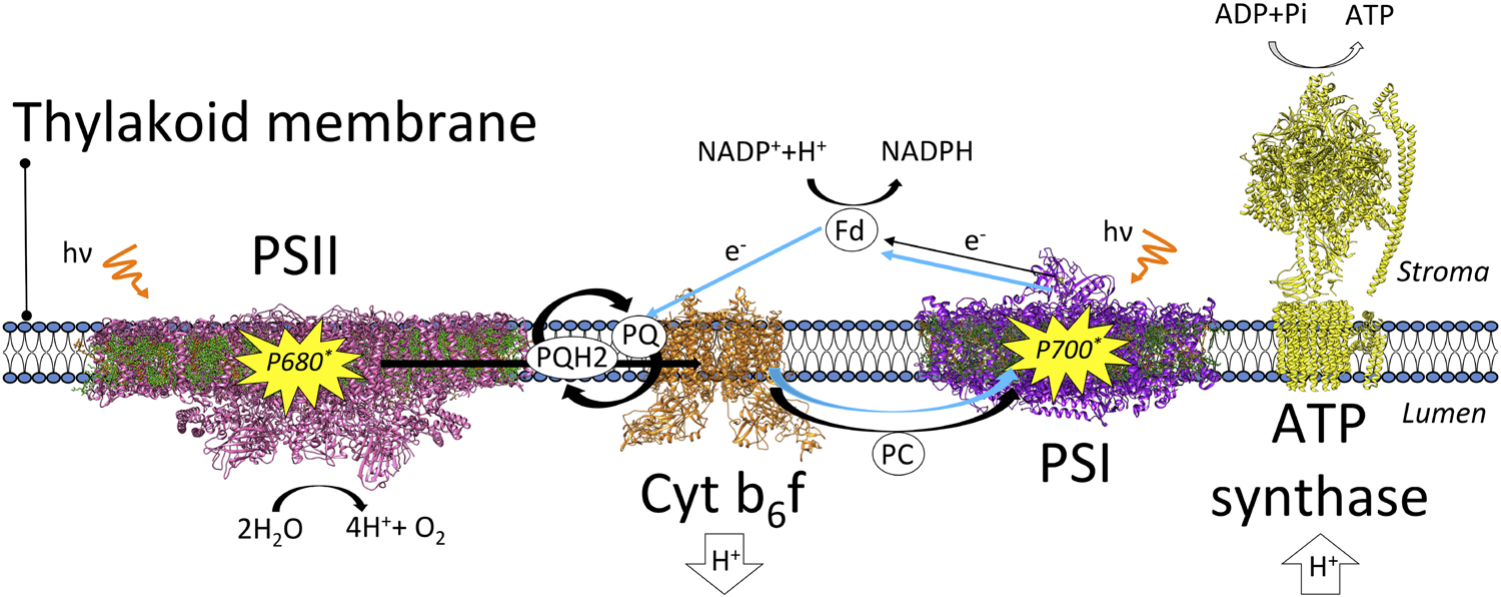
Schematic of the thylakoid membrane and of the light reactions. A simplified scheme of the linear and cyclic electron transfer pathways (in black and cyan solid arrows, respectively) is represented together with the involved photosynthetic subunits. Each complex is labeled in the figure, together with the charge transport pathways and the cofactors and proteins involved. The primary donors of PSII and PSI are represented in yellow. The structures for PSII, PSI, and Cyt b_6_f are taken from plants (Qin et al. 2015; Wei et al. 2016; Malone et al. 2019), the one for ATP from a bacterium (Morales-Rios et al. 2015). The thylakoid membrane is shown in blue

A simplified scheme of the functional organization of photosynthetic complexes within the thylakoid membrane is shown in Fig. 1. Two modes of electron transfer pathways take place and are defined as linear and cyclic electron flow, which we here briefly introduce one after the other. During linear electron flow, the excitation energy is first transferred from LHCs to the reaction center Chls (P680) situated in the core of PSII, where charge separation occurs. After charge separation, an electron is donated from P680 to a pheophytin and then to a plastoquinone (PQ) molecule bound to the complex in the so-called *Q*_A_ site. Electrons removed from water on the luminal side of the membrane are used to reduce P680+ . From the *Q*_A_ site, the electron is then transferred to a PQ molecule in the *Q*_B_ site. At this site, *Q*_B_-PQ after a second turnover of reduction accepts two protons from the stroma and detaches from the site in the form of plastoquinol (PQH_2_). This molecule diffuses through the membrane until it reaches the Cytochrome b_6_f complex. Here, the electrons from PQH_2_ are donated through a cycle of reactions (Q-cycle) to plastocyanin (PC), a luminal redox protein that then diffuses to PSI. During the Q-cycle, protons are also released into the lumen. In PSI, after Chl excitation has been funneled from the peripheral antennae to the reaction center (P700), charge separation produces an electron which is transferred via several cofactors present in PSI to ferredoxin (Fd), a small protein located on the stromal side of the membrane, while PC reduces back P700+. From Fd, electrons are donated to the NADP reductase (FNR) to produce NADPH, thus concluding the linear electron flow. The cyclic electron flow leads to the build-up of a proton gradient across the thylakoid membrane and operates at the level of Cyt b_6_f and PSI. The overall proton gradient built by both transport processes finally drives the ATP synthase to produce ATP. For a more exhaustive introduction to linear and cyclic electron flow and, more in general, to the light reactions, we refer the reader to the following reviews and textbooks (Joliot and Joliot 2006; Rochaix 2011; Blankenship 2014; Nawrocki et al. 2019).

In general, the thylakoid is a highly dynamic system in terms of structures, organization, composition, and functionality: the transport processes involved in the light reactions are only a part of the “dynamic processes” occurring in the thylakoid. Indeed, because everyday sunlight quality or quantity can change suddenly and irregularly, photosynthetic organisms need to respond to these changes via short-term and long-term acclimation strategies. These mechanisms are used to optimize the usage of light and to balance the amount of excitation in the thylakoid to prevent photooxidation. As a consequence, the structure and composition of the photosystems are dynamically regulated in response to changes in light conditions (Allen 1995; Kargul and Barber 2008; Chen et al. 2010; Niyogi and Truong 2013). On the one side, changes in the spectral quality of light lead to responses such as migration of LHCs from PSII to PSI or vice versa (so-called state transitions), changes in protein expression, or synthesis of pigments with an absorption cross section matching the available solar spectrum (Melis 1991). On the other side, an increase of irradiance causes activation of reversible photoprotective mechanisms to avoid photooxidation. Structural changes of photosynthetic subunits, specifically of the LHCs, regulate the fastest of such photoprotective responses (Müller et al. 2001; Ruban et al. 2012). In plants and algae, these conformational changes are caused by the light-dependent acidification of the thylakoid lumen (Li et al. 2009; Tokutsu and Minagawa 2013; Liguori et al. 2013, 2019; Gan et al. 2014; Dinc et al. 2016; Kondo et al. 2017), while in cyanobacteria structural changes in stress-related protein complexes are triggered directly by light (Kirilovsky 2007). The structural changes of pigments and proteins create quenching sites that shorten the excited state lifetime of the LHCs (photoprotective quenching), this way preventing accumulation of dangerous oxidizing species (Ruban et al. 2012).

Overall, this means that the single photosynthetic subunits continuously interchange among different conformations (e.g., via the photoprotective switch (Ruban et al. 2012), see above) and organizations (e.g., via state transitions (Melis 1991), see above). At a smaller scale, also the pigments can functionally change their structure and cofactors can move between different binding sites (e.g., in the case of the diffusion of PQ (Kirchhoff et al. 2000), see above). Thus, photosynthetic complexes and the thylakoid as a whole exist in a variety of states (Valkunas et al. 2012; Johnson and Wientjes 2019). The ensemble of states in which a single photosynthetic subunit or a complex of them can be found translates into the *conformational space* of the protein*.* In the specific case of chromophore-binding systems, the conformational landscape correlates not only with the protein *energetic landscape* but also with the optical properties of the pigment-protein complex. An example is the different conformations of the LHCs which correlate with different spectral properties (Krüger et al. 2011; Valkunas et al. 2012; Liguori et al. 2015). The various states in which photosynthetic systems can be found are separated by energy barriers: the higher the energy barrier, the less likely it is for a protein or a cofactor to change their conformation. In the case of single proteins, an associated conformational landscape with low energy barriers defines them as *disordered* systems. Via single molecule spectroscopy (SMS) it has been shown that LHCs have an inbuilt ability to switch reversibly between states more and less quenched and, therefore, between a variety of conformations, which has led LHCs to be defined as disordered systems (Krüger et al. 2010, 2011; Valkunas et al. 2012; Tian et al. 2015; Schlau-Cohen et al. 2015; Mascoli et al. 2019). Changes of the physiological conditions (e.g., nutrients, irradiance, spectrum, temperature, etc.) steer the photosynthetic subunits among different states in the landscape, this way controlling their conformation as well as their organization (Ruban et al. 2012; Valkunas et al. 2012).

To understand the molecular mechanisms of the light reactions, it is an invaluable asset to use a tool that can reconstruct the ensemble of possible conformations, reorganizations, interactions, and movements taking place within the thylakoid. In the past years, several high-resolution structures of the main photosynthetic complexes active in the first steps of photosynthesis (LHCs, PSII, and PSI) have been obtained via X-ray crystallography and cryo-electron microscopy (Pan et al. 2011; Umena et al. 2011; Fan et al. 2015; Qin et al. 2015, 2019; Wei et al. 2016; Su et al. 2017, 2019). These structures are in many cases a superposition of multiple conformations of these complexes and, therefore, lack information of the single states of the system. In addition, often, information on the structural response of the complexes to different physiological conditions is missing.

**Fig. 2:**
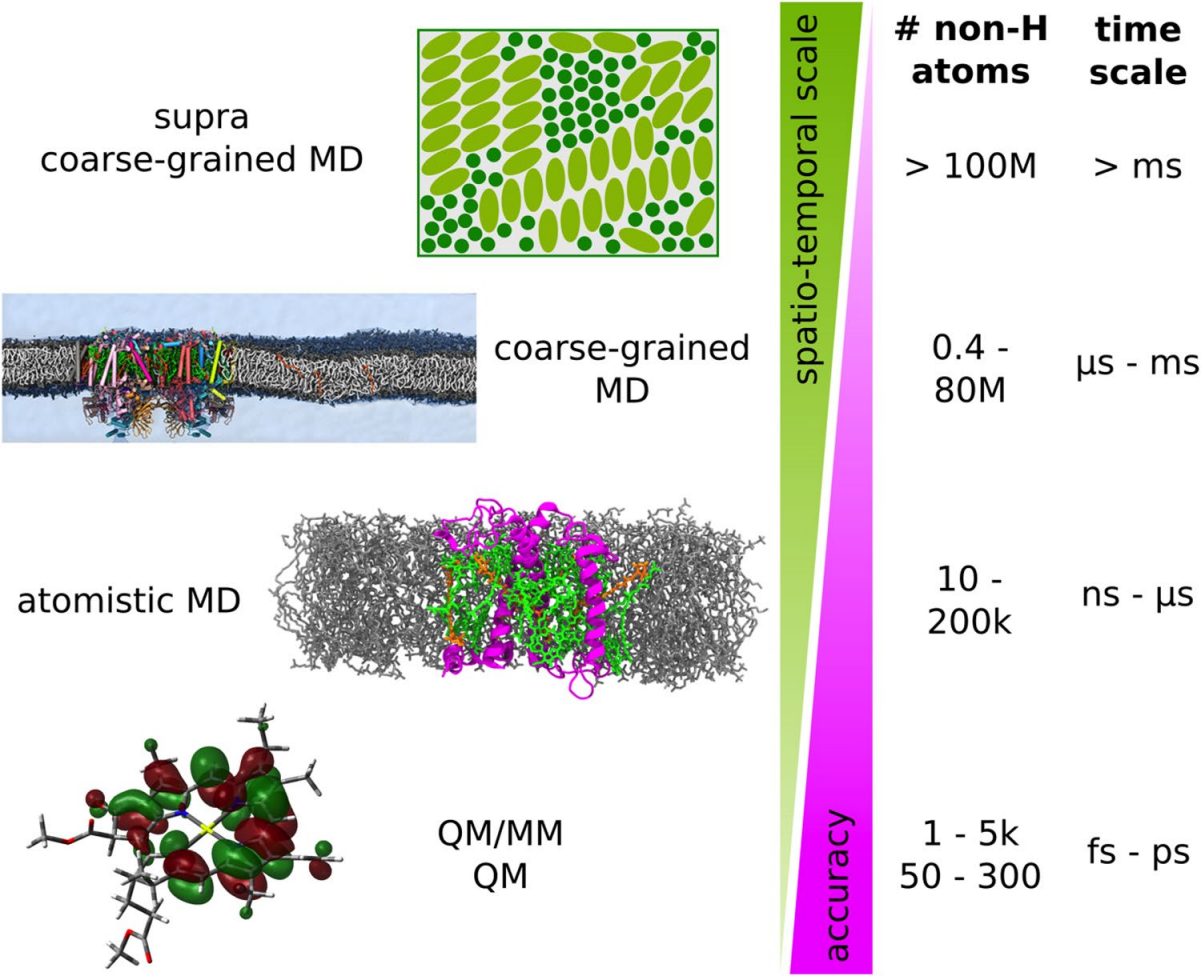
Simulation techniques with different spatio-temporal resolution illustrated using examples from photosynthesis research. With increasing system size, longer simulation times are required which entails a decrease in achievable accuracy. Typical ranges of system size in terms of number of non-hydrogen atoms and simulation time are indicated on the right. The main focus of our review is on atomistic and coarse-grained MD simulations; a glimpse on the other simulation techniques depicted here is provided in Sect. 4 at the end

Already since the late 1970s, molecular dynamics (MD) simulations are helping to characterize the dynamics of biological systems in silico with femtosecond and atomistic resolutions (Thiel and Hummer 2013). MD simulations allow sampling the conformational space of large biomolecular systems, providing information about the motion over time of every single atom. Pioneering work in the field of MD applied to photosynthesis was performed by Schulten’s group that, in the last decades, studied several light-harvesting systems with a focus on understanding the role of pigment dynamics and of the environment on tuning the spectral properties of the complexes (Schulten and Tesch 1991; Treutlein et al. 1992; Damjanović et al. 2002; Perilla et al. 2015). Due to limitations in the computational power at the time, only few tenths of ns were generally accessible for such large systems. As it will be shown in this review, recent progress in the development of dedicated force fields (see Sect. 2.1) for photosynthetic systems and the continuously increasing computational power allowed the field of MD applied to photosynthesis to explore in more detail increasingly longer timescales and larger sizes. The large range in spatio-temporal scale that is important for photosynthetic processes constitutes a major challenge to its computational treatment. Figure 2 shows that the system size ranges from single molecules—e.g., chromophores like Chls—, single proteins, protein supercomplexes, up to large assemblies of protein supercomplexes. With increasing system size, the required simulation time increases which entails a decrease in accuracy. In quantum mechanics (QM) and QM/molecular mechanics (MM) calculations, electronic and nuclear degrees of freedom are considered. As explained below in Sect. 4.2, QM/MM methods have been successfully used in the characterization of energy transfer and early electron transfer processes. This is not feasible for larger systems thus only the nuclear degrees of freedom are taken into account in atomistic MD simulations. For even larger systems, only simulations at the coarse-grained (CG) MD level provide reasonable simulation time. A diverse selection of photosynthetic complexes and processes has been successfully investigated on a wide range of spatio-temporal scales in the recent years by atomistic and CG classical MD, as presented below in Sects. 3.1, 3.2. If large parts of the thylakoids including numerous protein supercomplexes are simulated, supra CG methods are more suitable in which a protein is only represented by a small number of interaction sites. A brief overview of supra CG methods applied to photosynthesis is given below in Sect. 4.1.

This review aims to introduce the reader to the basic principles of MD simulations, their strengths, and limitations as well as their synergetic potential if employed in combination with experimental techniques. It is intended primarily for researchers in the field of photosynthesis who would like to strengthen their knowledge about MD or who are excited about applying MD methods. In particular, we focus on how atomistic and CG MD has been used to model photosynthetic systems of different size and complexity. Moreover, we give an overview of standard and non-standard approaches that have been used by the photosynthetic community so far. Before concluding, we provide a brief view on the techniques beyond atomistic and CG MD depicted in Fig. 2. The review primarily focuses on classical MD studies on oxygenic photosynthetic organisms. Anoxygenic photosynthetic organisms like purple bacteria have been studied in great detail computationally using mostly MD simulations in combination with QM methods. Because this goes beyond the scope of the current review, they are partly referred to in the last section of this review, which includes selected examples of combined QM/MM studies.

## Molecular dynamics principles

In biophysics, MD simulations are used to model small-scale biological systems (for example protein-membrane systems) as an ensemble of classical particles and to follow their dynamics enclosed in what is called a *simulation box*. This is done by analyzing the trajectory (coordinates and velocities) generated by the motion of each particle in the simulation box starting from a known set of initial positions, e.g., the protein coordinates from a high-resolution crystal structure. In this section, we review the basic concepts behind MD and explain how to set up and run an MD simulation.

### Interactions among atoms: what is a force field

In MD, each molecule is approximated as a system of classical point particles, or *interaction sites*. Depending on the chosen resolution, such particles may represent atoms or groups of atoms. The motions of the particles are obtained by solving the classical Newton equations for the system. The forces acting on the particles are computed over time and depend on the particle positions and the total potential energy of the system (*V*_tot_). Electrons are treated adiabatically which means that electronic degrees of freedom are not explicitly taken into account. The particles represent the properties of nuclei evolving on a Born–Oppenheimer potential energy surface. This also implies that molecules are studied in their electronic ground state. *V*_tot_ of the simulated system contains the bonded and non-bonded potential energies:

1 $$V_{{{\text{tot}}}} = V_{{{\text{bonded}}}} + V_{{\text{non-bonded}}}$$

*V*_bonded_ is the sum of the potential energy associated with chemical bonds, bond angles, and torsional angles (dihedrals) between groups of, respectively 2, 3, and 4 particles. The mathematical description of the bonded potentials can be slightly different for the different models which are available, but typically, the bonded potentials are modeled either via harmonic potentials (*V*_bond_ and *V*_angle_) or via cosine-based functions (*V*_dihedral_), as described by the following equations:

2 $$V_{{{\text{bonded}}}} = \; V_{{{\text{bond}}}} + \; V_{{{\text{angle}}}} + \; V_{{{\text{dihedral}}}} + \; V_{{{\text{improper\,dihedral}}}}$$

where

3 $$V_{{{\text{bond}}}} (d_{ij} ) = \frac{1}{2}K_{b} (d_{ij} - d_{{\text{b}}} )^{2}$$

4 $$V_{{{\text{angle}}}} \left( {\vartheta_{ijk} } \right) = \frac{1}{2}K_{{\text{a}}} ({\cos}\vartheta_{ijk} - {\cos}\vartheta_{{\text{a}}} )^{2}$$

5 $$V_{{{\text{dihedral}}}} (\phi_{ijkl} ) = K_{{\text{d}}} (1 + {\cos}(n\phi_{{{\text{ijkl}}}} - \phi_{{\text{d}}} ))$$

6 $$V_{{{\text{improper}} \,{\text{dihedral}}}} (\varphi_{{{\text{ijkl}}}} ) = \frac{1}{2}K_{{{\text{id}}}} (\varphi_{ijkl} - \varphi_{{{\text{id}}}} )^{2}$$

**Fig. 3:**
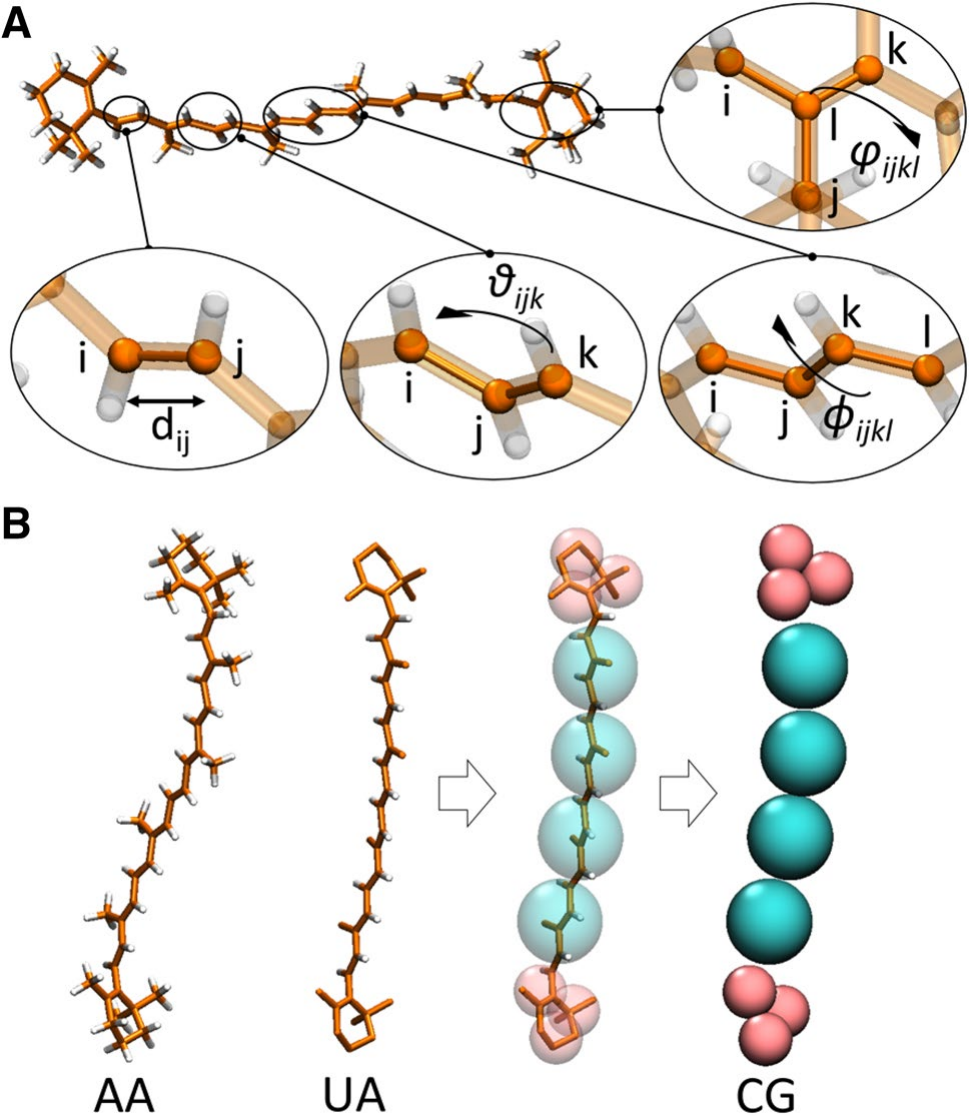
Bonded potential terms and force field resolutions. **A** Representation of the principal variables inside the bonded potential terms in the example of a betacarotene molecule: the variables are distance (${d}_{ij}$), angle (${\vartheta }_{ijk}$), dihedral angle (${\phi }_{ijkl}$), and improper dihedral angle (${\varphi }_{ijkl}$), and the different indices i, j, k, l refer to different interaction sites. **B** A betacarotene molecular structure represented at different levels of resolution: AA (all-atom resolution), UA [united-atom resolution, in particular in the case of GROMOS (de Jong et al. 2015)] and CG [coarse-grained resolution, in particular in the case of Martini (de Jong et al. 2015)]. The arrows indicate how the atoms of a UA-type of structure are grouped (“mapped”) into a CG-one

A representation of each term and the meaning of each variable are reported in Fig. 3A. The bonded potential *V*_bond_ describes the covalent bond stretching between 2 atoms. The angle potential *V*_angle_ carries information about the bending of 3 particles and is associated with three covalently bound particles. The dihedral potential *V*_dihedral_ describes the angle between the two planes formed by 4 covalently bound particles (Fig. 3A); where *n* represents the periodicity of the potential. When necessary, the improper dihedral potential $(V_{{{\text{improper}} {\text{dihedral}}}} )$ can be used to maintain the planarity of the molecule, for example, within rigid planar structures such as tetrapyrroles or along conjugated chains. All force constants (*K*_b_, *K*_a_, *K*_d_, and *K*_id_) are chosen to reproduce the expected stiffness of the molecule. Together with the equilibrium constants $(d_{{\text{b}}} ,\vartheta_{{\text{a}}} ,\phi_{{\text{d}}} ,\;{\text{and}}\;\varphi_{{{\text{id}}}} ),$ they are established based on ab initio calculations which are often additionally refined to reproduce selected experimental observables (see below the details on how these parameters are derived and validated).

*V*_non-bonded_ describes the interaction between any pair *i*,*j* of particles. It is the sum of van der Waals, often modeled as a Lennard–Jones (*L*–*J*) potential (*V*_L–J_), and Coulomb (*V*_coulomb_) interactions:

7 $$V_{{{\text{L}} - {\text{J}}}} \left( r \right) = 4\varepsilon_{ij} \left[ {\left( {\frac{{\sigma_{ij} }}{r}} \right)^{12} - \left( {\frac{{\sigma_{ij} }}{r}} \right)^{6} } \right]$$

8 $$V_{{{\text{coulomb}}}} \left( r \right) = \frac{{q_{i} q_{j} }}{{4\pi \varepsilon_{0} \varepsilon_{r} r}}$$

where *ε*_*ij*_ is the depth of the potential well of *V*_L–J_, and *σ*_*ij*_ is the distance between the pair of particles at which the potential is zero. In *V*_coulomb_, *q*_*i*_ and *q*_*j*_ are the charges of two different particles, while *ε*_o_ and *ε*_r_ are, respectively, vacuum permittivity and the relative dielectric constants. In both potential terms, *r* is the distance between the two particles.

The set of all the information needed to build up the potential energy terms of a system of particles is called the *force field* (FF). It consists of the list of functional forms and parameters for all bonded potentials as well as for the non-bonded terms. Depending on the spatial resolution describing the molecules in the system, FFs are divided in atomistic and coarse-grained FFs:

#### Atomistic force fields

For small proteins and in general systems of few tens of thousands of atoms, there is the possibility to model them accurately and for long simulated timescales (up to the μs range) using an all-atom force field, that is to say that all the atoms of the system are taken into account as individual particles when solving the equations of motion. However, in the effort to sample as much conformational space as possible and, therefore, to reduce the computational cost related to sampling, it is possible to simplify the system with the so-called united-atom (UA) FFs (Jorgensen et al. 1996; Oostenbrink et al. 2004; Yang et al. 2006). In this approximation, all non-hydrogen atoms are explicitly taken into account but some of the hydrogens of non-polar groups are included implicitly in the particle representing the non-hydrogen atom of the corresponding group (see Fig. 3B). This way, the number of particles is reduced and some of the fastest vibrational motions of the molecule are suppressed, which reduces the computational cost of the simulation.

Currently, several all-atom and UA FFs are available and validated for all amino acids, several lipid classes, and different solvents (Oostenbrink et al. 2004; Zhu et al. 2012; Nerenberg and Head-Gordon 2018). Importantly for the photosynthetic community, nowadays several of these FFs have also been derived and validated for photosynthetic pigments and cofactors: most all-atom FFs (such as CHARMM and Amber) have been validated by comparing simulated properties against ab initio calculations (Damjanović et al. 2002; Ceccarelli et al. 2003; Karki and Roccatano 2011; Cerezo et al. 2013; Guerra et al. 2015; Adam et al. 2018; Kim et al. 2018). In the case of the Amber force field for Cars (Prandi et al. 2016), this set of parameters has been specifically validated to reproduce selected spectral properties. The UA FF available for the pigments, cofactors, and lipids associated to photosystem II (PSII) and the LHCs (López et al. 2013; van Eerden et al. 2015; de Jong et al. 2015; Liguori et al. 2015) have been developed based on the building blocks of GROMOS. Respecting the guiding rules of this FF (Oostenbrink et al. 2004, 2005; Schmid et al. 2011), they have been validated against experimental data.

#### Coarse-grained force fields

To be able to simulate larger biomolecular ensembles for longer simulation times, the resolution of the molecular representation has to be reduced. Metaphorically speaking, it corresponds to a zooming out of the simulation box containing the biomolecular ensemble. CG FFs are used for this step (Saunders and Voth 2013; Noid 2013; Ingólfsson et al. 2014). They do not describe every atom explicitly but typically group 3–6 non-hydrogen atoms together in one CG interaction site (see Fig. 3B). These are often called CG beads. The resulting lower number of particles in the simulation box reduces the computational cost. A CG bead imitates the average properties of the atoms it represents. There are two major strategies to generate CG FFs called bottom-up and top-down, which tackle the FF parametrization from two opposing sites. While in a bottom-up approach structural properties are the key parametrization targets, in a top-down approach experimental ensemble properties are the major parametrization targets. Often, a combination of both strategies is applied during the parametrization. There exist various CG FFs, e.g., the Martini FF (Marrink et al. 2007; Marrink and Tieleman 2013), the SIRAH (south-American initiative for a rapid and accurate Hamiltonian) FF (Machado et al. 2019), the ELBA (electrostatics-based) FF (Orsi and Essex 2011), and the SPICA FF (DeVane et al. 2009; Seo and Shinoda 2019), which describe water explicitly. Other CG models like e.g., the CgProt FF (Hills et al. 2010), the PLUM FF (Bereau et al. 2014), or the Dry Martini FF (Arnarez et al. 2015), treat the water environment implicitly.

The currently most-widely used CG FF operating at almost atomistic resolution is the Martini FF (Marrink et al. 2007; Marrink and Tieleman 2013). It groups approximately four non-hydrogen atoms in one CG bead based on their physico-chemical characteristics. For example, it treats a functional group like an ester group always as one unit and thus combines it to one bead. The non-bonded interactions of these building blocks are obtained by comparison to experimental partitioning data. The bonded terms are chosen to optimally represent the molecular structure usually obtained from atomistic reference simulations. The selection of the building blocks based on the chemical nature of the grouped atoms allows combining multiple classes of biomolecules like lipids (Wassenaar et al. 2015), proteins (Monticelli et al. 2008; de Jong et al. 2013), sugars (López et al. 2009), DNA (Uusitalo et al. 2015), and RNA (Uusitalo et al. 2017) in one simulation.

Besides the lower number of particles, CG simulations come with another advantage with respect to their computational efficiency: the potential energy landscape is smoothened compared to atomistic FFs which allows the use of a larger time step (see Sect. 2.2). One effect is that the accessible total simulation time increases with respect to a limited computational time. In addition, the smoothened potential energy landscape facilitates the transition between different local minima. However, it entails also limitations: the already-mentioned lower resolution inherently leads to a loss of directed interactions which are mediated by the defined orientation of specific atoms in one CG bead. An important case is represented by hydrogen bonds, which play a key role in stabilizing the secondary and tertiary protein structure. To compensate for their lack in CG models, they are often taken into account by additional bonded interactions in the so-called elastic network (Atilgan et al. 2001; Periole et al. 2009) or Gō-like models (Taketomi et al. 1975; Poma et al. 2017; Thallmair et al. 2019). In addition, the time scale of the simulations does not correspond to real time anymore but is faster by a factor of approximately 3–10 due to the smoothened energy landscape (Marrink et al. 2007). Another limitation is the introduction of artificial energy barriers upon dimerization of molecules because one CG water bead represents multiple water molecules (Alessandri et al. 2019). Moreover, the bond lengths and the bonded force constants should remain in the range for which the CG FF was parametrized. Too short bond lengths or too weak force constants can artificially increase the non-bonded interactions (Alessandri et al. 2019).

### Time-evolution: integrators

In order to obtain information about the time evolution of the (bio)molecular system, the numerical integration of the equations of motion is necessary:

9 $$v = - \frac{{{\text{d}}r}}{{{\text{d}}t}},\,\, a = - \frac{{{\text{d}}v}}{{{\text{d}}t}}$$

With $v$ being the velocity, *r* the position, *t* the time, and *a* the acceleration. Both equations can be combined to describe the time evolution by one time step ∆*t* (Frenkel and Smit 2002):

10 $$r\left( {t + \Delta t} \right) = r\left( t \right) + v\left( t \right)\Delta t + 0.5a\left( t \right)\Delta t^{2}$$

The calculation of the velocity $r\left( {t + \Delta t} \right)$ for the next time step requires the acceleration *a*, which can be obtained from the force *F*:

11 $$a = \frac{F}{m} = - \frac{{{\text{d}}V_{{{\text{tot}}}} }}{{{\text{d}}r}}\frac{1}{m}$$

With *m* being the mass and *V*_tot_ the total interaction potential (Eq. 1). The force is calculated as the negative derivative of the potential energy, which depends on the FF parameters and the actual atomic positions. To perform the numerical integration of the equations of motion, the equations have to be solved using small discretized time steps ∆*t*. With the atomic positions at *t* + ∆*t* at hand, the new forces and new velocities can be calculated which are then used to propagate the system forward in time by ∆*t*. In atomistic simulations, where fast motions of atoms such as the hydrogen atoms are taken into account, ∆*t* must be of the order of a few fs, making it computationally unfeasible to obtain long trajectories (> μs). In the case of CG simulations, ∆*t* is typically on the order of a few tens of fs.

There are different integrators available for MD simulations. The simplest one, which is given in Eq. (10), is the Euler integrator (Frenkel and Smit 2002) but it is practically not employed. Commonly used integrators are the leap frog (Verlet 1967), and the velocity Verlet integrator (Swope et al. 1982); the latter is an improved version of the Verlet integrator. They have higher accuracy compared to the Euler integrator. However, their purpose in the MD simulations is the same—they propagate the system in time. For more details, the interested reader is referred to standard MD textbooks (Frenkel and Smit 2002; Berendsen 2007).

### Work flow of an MD simulation

**Fig. 4:**
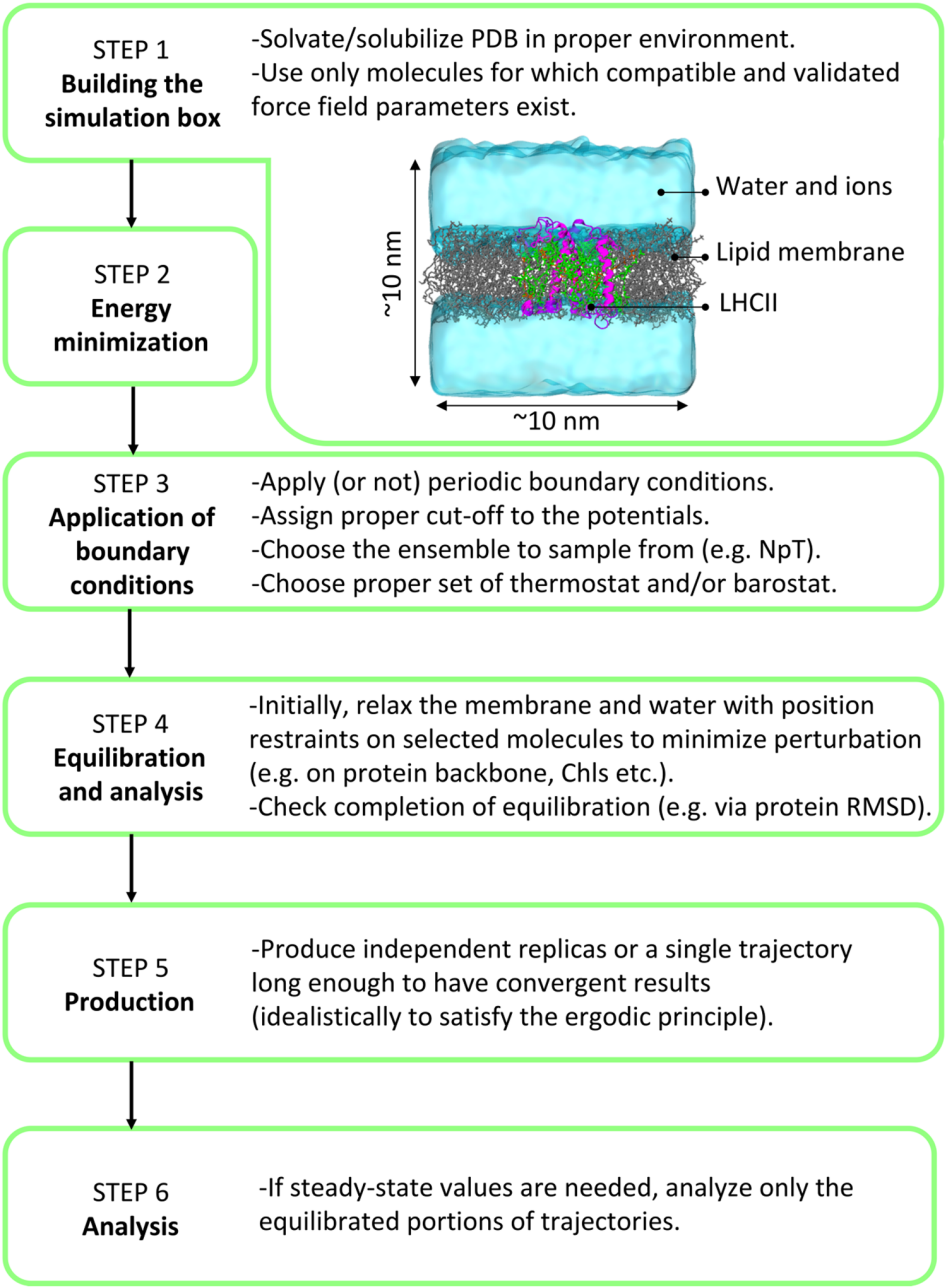
Workflow of an MD simulation, as described in Sect. 2.3. The sequential steps are reported together with the associated main points that need specific attention. In the inset, an example of a simulation box for LHCII embedded in a model membrane is reported with water in cyan, lipid membrane in gray, protein in magenta, Chls in green, and Cars in orange

Here, we summarize the main steps necessary to set up and run an MD simulation. A summary of the workflow is reported in Fig. 4.

#### Building the simulation box

A simulation box must contain only molecules that can be described by FF parameters previously validated and compatible with each other, i.e., for the protein, the pigments and any cofactor, the lipids, the solvent, and the ions.

After obtaining the coordinates of the photosynthetic system of interest (e.g., an LHC), e.g., from a database such as the Protein Data Bank (Bernstein et al. 1977), the system should be (1) checked for missing residues or atoms. In case of gaps in key regions of the structure, these gaps should be filled via sequence homology modeling, e.g., as done for PsbS (Liguori et al. 2019), by using information from other resolved structures of similar proteins, e.g., as done in the case of missing Chls’ phytol tails (Liguori et al. 2015), or by predicting the structure of the missing amino acid sequence, e.g., as done in the case of LHCII (Thallmair et al. 2019); (2) before starting the MD simulations, the photosynthetic system must be embedded in a meaningful environment, i.e., a lipid bilayer or a detergent micelle in the case of a membrane protein, water in the case of water-soluble proteins, etc. In most cases and in particular if the simulation will be run at an atomistic resolution, the embedding system should have been pre-equilibrated before insertion of the protein complex. The obtained protein-surfactant system (or a water-soluble protein) can then be solvated with water molecules. A different solvent can also be used (if compatible FF parameters are available). Counterions should be added if necessary to neutralize the charge of the system and salt can be added to reproduce physiological conditions. Nowadays, dedicated setup tools like, e.g., the CHARMM-GUI website (Wu et al. 2014; Jo et al. 2017), or the programs moltemplate.sh (www.moltemplate.org), HTMD (Doerr et al. 2017), and insane.py (INSert membANE) (Wassenaar et al. 2015) are available. They enable the user to generate starting conformations for complex biomolecular systems comparably straightforward.

#### Energy minimization

The preliminary simulation box potentially contains clashes between atoms that must be removed before running the MD simulation. These clashes are energetically extremely unfavorable because of the steep gradient of the repulsive part of the L–J potential (see Sect. 2.1). Clashes can be removed by running an *energy minimization, i.e.,* an algorithm which, through a series of steps, optimizes the positions of each atom (or CG bead) based on the potential terms associated with the chosen FF. Different minimization methods exist mostly employing a gradient descent optimization algorithm. After the energy of the system has been minimized, MD simulations can be run.

#### Choice of the boundary conditions

To further apply a control on the physiological conditions of the simulation, the MD integrator (Sect. 2.2) must be run with additional tools that restrain the system: indeed, to macroscopically reproduce the target experimental temperature and/or pressure within the simulation, a so-called *thermostat* and/or a *barostat* must be applied. Whether one or both of these tools must be applied depends on the experimental conditions that should be modeled and, consequently, on the statistical ensemble that should be simulated. For example, in case of a canonical ensemble (NVT) in which the number of particles in the box (*N*), the volume (*V*) and the average temperature (*T*) are constant, a thermostat must be used. In this case *V* and *N* are set at the start of the simulation and left unvaried during the trajectory. T cannot be fixed because it is an average macroscopic property derived from the total kinetic energy of the system and, as such, it should experience fluctuations. Similarly, in the case of an isothermal-isobaric ensemble (NpT), the average pressure (*p*) must be constrained and a barostat must be used in combination with a thermostat. Several methods exist for ensuring that *T* and/or *p* oscillate around the target values (Andersen 1980; Parrinello and Rahman 1981; Nosé and Klein 1983; Berendsen et al. 1984; Hoover 1985; Bussi et al. 2007): in the first case, the thermostat regulates the fluctuations of the velocity distribution and, consequently, the macroscopic average temperature of the box. In the latter case, the barostat regulates the fluctuations in the volume of the simulation box and, consequently, the macroscopic average pressure. Special barostat settings are applied for membrane systems: The semi-isotropic pressure coupling scheme allows to regulate the pressure in the plane of the membrane—typically the *x*,*y* plane—independently from the pressure in the aqueous phase which is controlled along the membrane normal—the *z* axis (Smith et al. 2019). This ensures a homogeneous pressure within the membrane as well as in the water phase without the need of precisely adjusting the ratio of lipid and water molecules during the setup. Each thermostat and barostat algorithm has its own limits and advantages and the choice of the specific tool to be used must be done thoughtfully (Frenkel and Smit 2002; Hünenberger 2005).

Another boundary condition that must be applied concerns how to treat the edges of the simulation box, which has a finite size. Surface effects can be minimized by replicating the simulated box in all the dimensions, applying the so-called *periodic boundary conditions*. These replicate boxes are called *image cells*. Cut-offs in the non-bonded pair potentials must be applied to avoid interactions between a particle in the primary simulation box and the same particle in the image cells. Finally, by applying what is called a *minimum image convention*, each particle is allowed to interact only with the closest images of all the other particles present in the system.

#### Equilibration of the system and production

The energy minimization produces the set of starting coordinates to be used for the MD simulation. The initial velocities are (generally) randomly extracted from a Maxwell–Boltzmann distribution of velocities associated with the target temperature.

The initial portion of simulated time must be then allocated to the *equilibration* of the system. Various strategies exist on how to equilibrate a biomolecular ensemble starting from a crystal structure. In most cases, an NpT simulation is suitable to simultaneously relax the system's temperature and pressure. In more delicate cases, it might be necessary to start the equilibration phase with a brief NVT simulation to first ensure a proper relaxation of the system temperature, followed by an NpT simulation. In any case, both temperature and pressure should be monitored to ensure that the average target values have been reached without further drifts.

Importantly, additional time is required to equilibrate other properties of the system. For example, for MD simulations with lipid membranes, the membrane should reach stable values of e.g., thickness, area per lipid, hydration, etc., that are expected for the chosen lipids. This equilibration usually takes on the order of hundreds of nanoseconds depending on the size of the membrane patch (Marrink et al. 2019). If proteins and cofactors are also present, as in the LHCs, additional care must be taken to ensure that, during the time needed to relax and equilibrate the solvent and the membrane, the initial high-resolution structure is not perturbed. A good practice to avoid this is to apply strong position constraints (on the order of 1000 kJ mol^−1^ nm^−2^) on the atomic positions of the protein backbone (or *C*_alpha_ carbons) and on the pigments’ and cofactors’ parts whose structure is most critical to the spectral or functional properties of the complex during the first equilibration period (Ogata et al. 2013; Liguori et al. 2015, 2017). After the surrounding system has reached equilibrium, the constraints on the photosynthetic complex can be removed.

To be noted, an additional period is finally mandatory to equilibrate the whole system. This equilibration can be monitored through different quantities, depending on the type of the analyses that will be run. Typical measures to monitor the equilibration process beyond temperature and pressure are the total, the kinetic, and the potential energy of the system, the root-mean-square deviation (RMSD) of proteins, the area per lipid or thickness for membranes, and the number of contacts between different lipids types for multi-component membranes. In general, every analysis run on the whole simulated trajectory shows after which time period the computed quantity has eventually reached a steady-state value. When the key properties of the system have reached a plateau, the system can be considered at equilibrium and all the analyses should be done on the equilibrated part of the trajectory, which is often referred to as the *production* part of the MD simulation.

### Limited sampling and non-standard MD simulations

If the MD trajectory is long enough, it is nowadays feasible to reach equilibrium for the simulated system with respect to processes taking place on the low µs time scale. However, in most cases, the system will not be simulated long enough to sample from an equilibrium distribution, meaning that the *ergodic principle* does not hold, i.e., the ensemble average of a quantity will not be equal to its average over the simulated time. To overcome this lack of sampling, it is essential to run multiple independent replicas of the same simulation. One possible way to obtain independent replicas is to assign different initial random velocities in each replica.

Enhanced sampling techniques provide an alternative way to achieve meaningful sampling of states or processes that take place beyond the common time scale of atomistic or CG MD simulations within a reasonable computing time. Typically, the goal is to sample regions of the potential energy surface that exhibit higher energy and are thus not often visited during a standard MD simulation. However, those regions can be important for biophysical and biochemical processes. One example is the sampling of transition pathways connecting different free energy minima that are separated by a significant barrier.

**Fig. 5:**
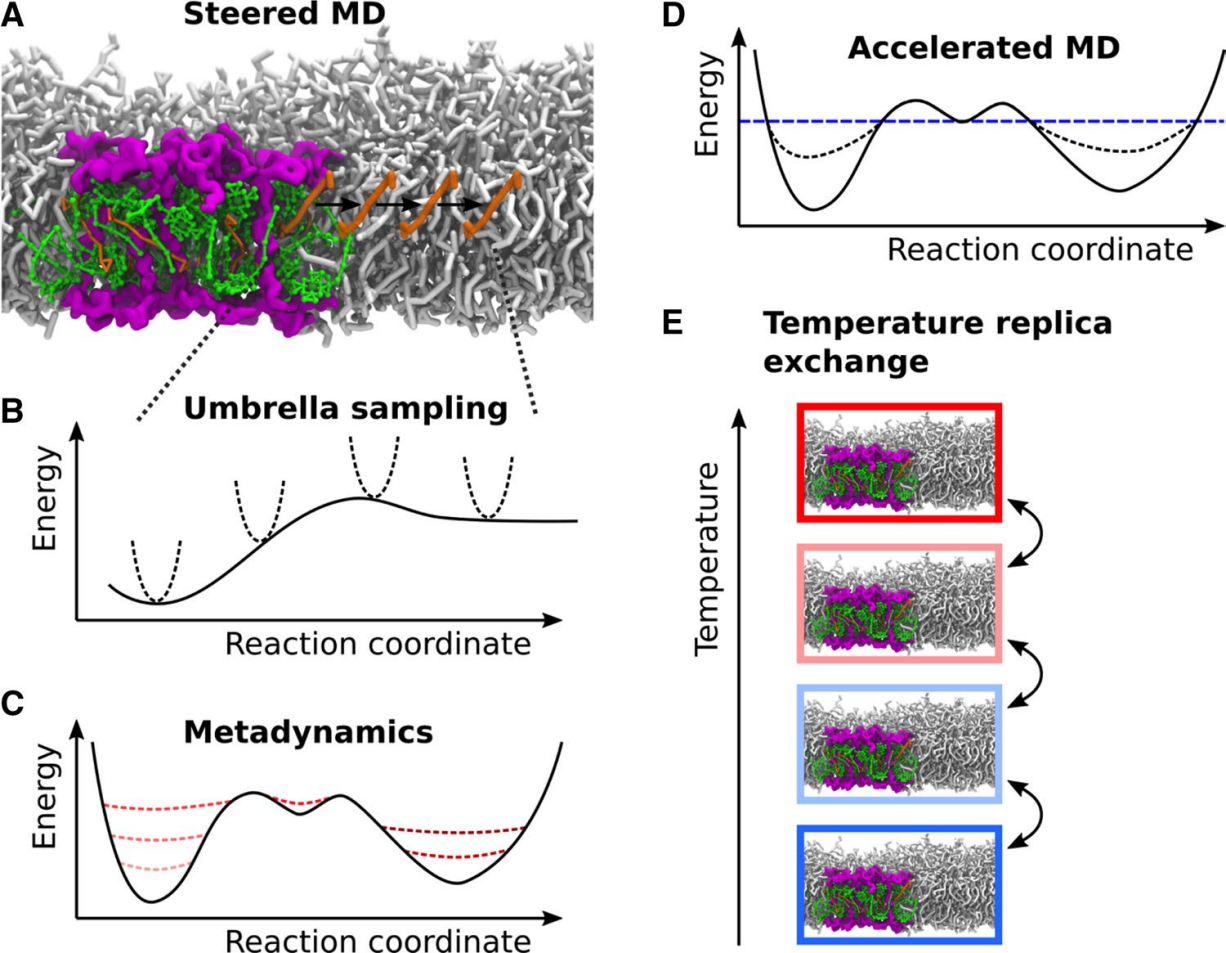
Schematic representation of selected enhanced sampling techniques. **A** Schematic visualization of steered MD. A force (black arrows) is applied to one carotenoid to steer its unbinding from the LHCII binding pocket into the membrane. **B** During umbrella sampling, a biasing potential represented by the dashed harmonic potentials fixes the system along the reaction coordinate. The solid line shows the original potential. **C** In metadynamics, the biasing potential is added in the regions which were already visited along the reaction coordinate. The dashed lines indicate the changing potential energy surface during time (from light to dark red). **D** In accelerated MD, the biasing potential is time-independent resulting in the potential depicted by the black dashed line. **E** During temperature replica exchange, the system is simulated at different temperatures. Configurations are exchanged if the selection criteria are met

Enhanced sampling techniques can be divided in (i) techniques that require a predefined reaction coordinate along which the sampling is enhanced, and (ii) techniques that do not require such a reaction coordinate. One example of the first group is umbrella sampling (Torrie and Valleau 1974; Kästner 2011). The approach requires knowledge of the coordinate along which the process of interest happens. An additional biasing potential is applied to drive the system step by step from one state to the other ensuring sufficient sampling along the whole path, as shown in Fig. 5B. Steered MD offers an alternative to guide the system along a reaction coordinate using external forces (Izrailev et al. 1999; Vassiliev et al. 2012). A representation of steered MD is given in Fig. 5A. Despite the irreversibility of the simulations, they provide information about pathways and offer a comparison to atomic force microscopy (AFM) experiments. Another approach to increase the sampling along a reaction coordinate is metadynamics (Laio and Parrinello 2002; Barducci et al. 2011). Here, a history-dependent biasing potential is added along selected reaction coordinates, the so-called collective variables. Thus, the biasing potential is updated during the MD simulation. The goal is to drive the system away from the regions of the potential energy surface that have been already visited during the simulation. A scheme of metadynamics is reported in Fig. 5C.

Accelerated MD belongs to the group of enhanced sampling techniques that do not require a predefined reaction coordinate (Voter 1997; Hamelberg et al. 2004; Miao and McCammon 2016). A time-independent bias potential, which only depends on the potential energy, is added to the latter if it is below a certain threshold value (see Fig. 5D). This facilitates exploring higher energy regions of the potential energy surface above the energy threshold, which are unaltered. Another technique not requiring any predefined reaction coordinate is temperature replica exchange (Sugita and Okamoto 1999; Miao and McCammon 2016). Multiple simulations at different temperatures, so-called replicas, are performed simultaneously and configurations are exchanged between replicas if certain conditions are met. The idea is to facilitate the transition of barriers at higher temperatures and to subsequently cool down the system after the barrier crossing by transferring the configurations to the replica at a lower temperature. A scheme for temperature replica exchange is reported in Fig. 5E. Lots of variations of the replica exchange method exist which are in general termed Hamiltonian replica exchange.

If sufficient sampling is achieved using one of the aforementioned enhanced sampling techniques, free energy differences between the states visited can be computed. Another option to calculate the free energy difference between two states provides thermodynamic integration (Straatsma et al. 1986; Abrams and Bussi 2013). Here, the change in free energy along a non-physical path can be calculated. But because the free energy is a state function, its difference between two states does not depend on the path connecting them. Thus thermodynamic integration allows, e.g., calculating the free energy change caused by single point mutations or by cofactor oxidation. Practical aspects of different free energy methods are summarized in this recent review (Hansen and van Gunsteren 2014).

## MD in photosynthesis: characterizing slow and fast motions in the thylakoid membrane

In this section, we will give an overview of how classical MD simulations have been applied so far in the field of photosynthesis: we will zoom-in by going from the slower dynamics at the large membrane scale to the faster dynamics of single photosynthetic subunits. Concomitantly, we will give an overview of how such computational studies relate to experimental evidence.

### Beyond the μs timescale: interplay between pigment-protein complexes and the lipid membrane

Both UA and CG FF parameters for all of the major glycolipids of the thylakoid membrane were released in 2013, with the publication of the Martini and GROMOS force field for glycolipids (López et al. 2013). In 2013, thylakoid lipids with atomistic resolution (General Amber force field, GAFF) were used to model PSII dynamics in a thylakoid membrane (Ogata et al. 2013). An extensive report of the behavior of lipids in the thylakoid membrane was published in 2015 (van Eerden et al. 2015). In this work, lipid membranes consisting of up to 2000 lipids, with simulation boxes as large as 25.5 × 25.5 nm in the lateral dimensions, were simulated up to 10 μs per system. Thylakoid membrane patches were modeled with the lipid composition of either plants or cyanobacteria, using compositions determined experimentally (Sakurai et al. 2006). The simulations were run mainly at the CG resolution (Martini FF) because of the slow phenomena under investigation, i.e., lipid mixing and lipid–lipid interactions. It was found that all lipid types distribute homogeneously within the membrane patch, with clusters detectable only at the nanoscale. This finding agrees with the even distribution of glycerolipids observed in thylakoids experimentally (Duchêne and Siegenthaler 2000). All lipid classes also showed to have a rather similar diffusion speed, in the order of 20–30 μm^2^/s at room temperature (however, this diffusion speed can be expected to be ~ 4 times slower in reality due to the faster Martini CG dynamics) (van Eerden et al. 2015). Due to its higher degree of saturation (Sakurai et al. 2006), cyanobacteria membrane resulted to be less fluid and also more ordered and thicker than the plant one. The simulations also showed that plant thylakoids tend to form an inverted hexagonal phase more likely compared to cyanobacteria. This polymorphism was attributed to the larger fraction of polyunsaturated fatty acids in the plant membrane (van Eerden et al. 2015). An inverted hexagonal phase was indeed observed in plant thylakoid membranes (Krumova et al. 2008), but the role of this phase in photosynthesis is still unclear.

Within the membrane, the dynamics of the photosystems and of the associated lipids and cofactors are also characterized by rather slow motions (μs-to-ms timescale). In 2017, MD simulations up to 100 μs at CG resolution (Martini) were performed both for the PSII dimer and monomer of cyanobacteria in a model thylakoid membrane, for a cumulative simulated time of about 0.5 ms (van Eerden et al. 2017a, b, c). Remarkably, the RMSD of the protein reported by van Eerden et al. does not reach a constant value before the first ~ 20 μs (van Eerden et al. 2017a, b, c). The RMSD of the protein backbone is a suitable probe for the equilibration of a simulated system (see Sect. 2.3) and, in this case, it indicates that a complex as large as PSII takes at least few tenths of μs to reach equilibrium in the membrane. Moreover, in a separate work (Van Eerden et al. 2017a), it is illustrated how even a substantial simulation time of ~ 85 μs is not yet long enough to obtain convergent results on the binding of lipids to the different monomeric subunits of PSII dimer. This strongly suggests that simulations should be run for at least several tenths of μs when investigating photosynthetic complexes of similar size and complexity to obtain statistically meaningful analyses, a timescale accessible to CG methods, but more hardly to atomistic simulations (Fig. 2).

MD can also be used to investigate the dynamics of associated molecules like e.g., cofactors in the complexes and in the membrane. For example, steered-MD simulations were used to compute the energetic cost of water diffusion inside PSII (Vassiliev et al. 2012) at atomistic resolution (Amber (Simmerling et al. 2002)). In this work, it was found that all the water channels inside PSII have an activation energy for water permeation higher than the one in lipid membranes (Hub and De Groot 2008), thus suggesting that in PSII internal water diffusion is regulated, which is beneficial to stabilize the oxygen-evolving cluster during turnover. Atomistic MD simulations combined with in silico mutational analysis were used to characterize the role of specific residues and protonation states in controlling the long-distance hydrogen-bond networks that connect the manganese cluster region to the lumen of the thylakoid (Guerra et al. 2018).

In another work, in agreement with previous isotope labeling experiments (Beisel et al. 2010), CG trajectories of PSII (van Eerden et al. 2017a, b, c) suggested that some β-carotene (BCR) can freely diffuse in and out of PSII. The mobility of BCR in the membrane is as high as the one of lipids (see above), and it was computed to be between ~ 30 and ~ 50 μm^2^/s, depending both on the atomistic force field used (respectively, GROMOS (de Jong et al. 2015) and OPLS (Jemioła-Rzemińska et al. 2005) FF) and the lipid types present in the membrane (DPPC (de Jong et al. 2015) vs POPC (Jemioła-Rzemińska et al. 2005)). As expected, the diffusion rates at CG resolution resulted to be about four times higher than the ones with atomistic force fields (de Jong et al. 2015). To the best of our knowledge, no experimental data are currently available on the diffusion coefficient of BCR in membranes. CG simulations of Chl a and b using specially designed Martini bead types showed a diffusion constant of approximately 0.1 µm^2^/s in DPPC model membranes, which is 7–8 times higher than in atomistic simulations (Debnath et al. 2015). Compared to DPPC, the observed Chl diffusion is two orders of magnitude lower. In addition, the Chls showed aggregation in the DPPC bilayer in agreement with fluorescence quenching experiments in DPPG micelles.

**Fig. 6:**
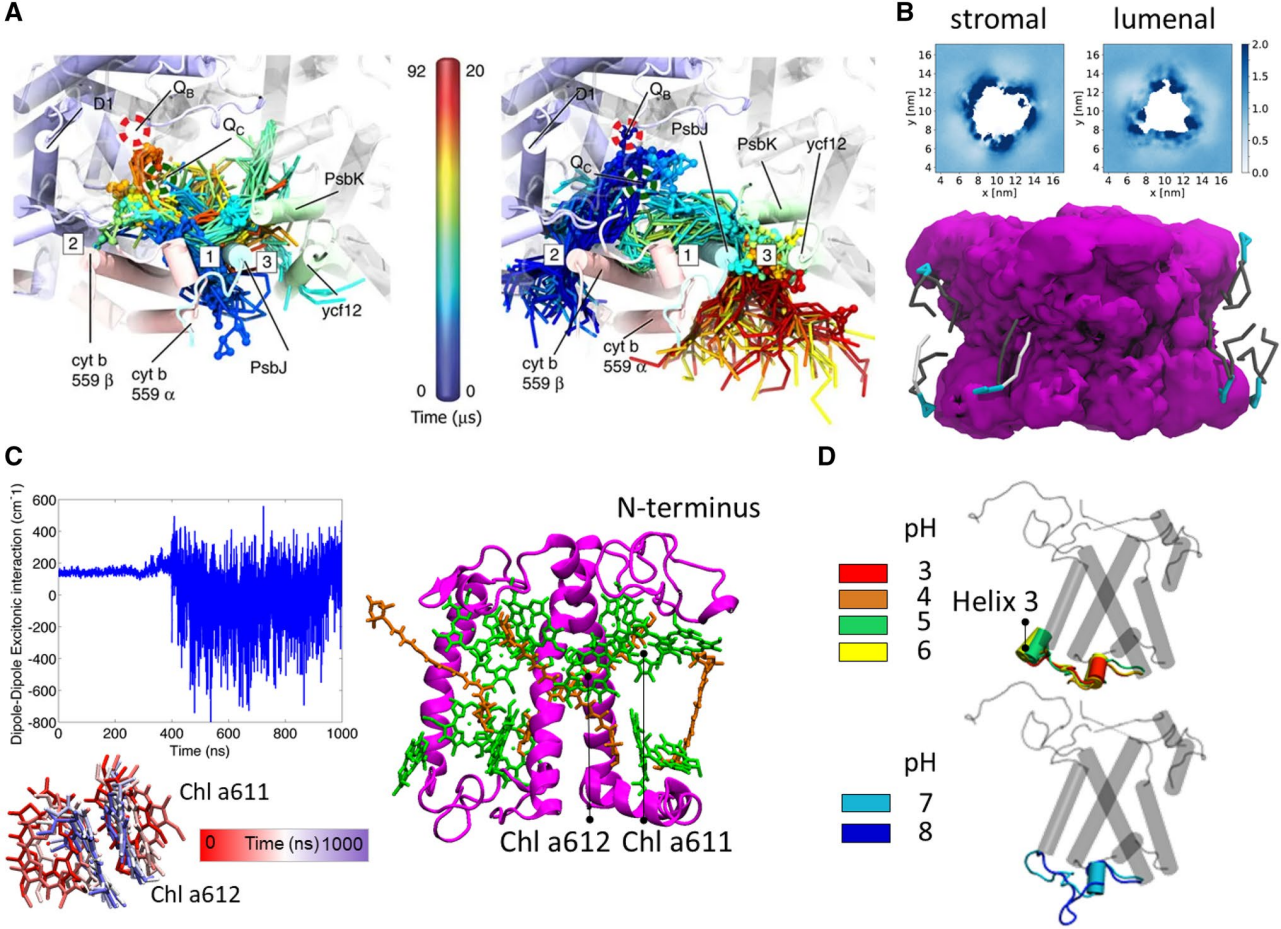
Examples of structural insights on the function of photosynthetic protein complexes via MD simulations. Selected results from the MD work of our groups are presented here: **A** Snapshots of diffusive entrance of PQ (left) and exit of PQH2 (right) of PSII reaction center. The red and green dashed circles indicate the *Q*_B_ and *Q*_C_ binding sites, respectively. Figure reproduced with permission from Ref. (Van Eerden et al. 2017b). **B** Relative MGDG density around an LHCII trimer in the stromal and lumenal leaflet of the thylakoid membrane (top). The average volume density (bottom, magenta surface) of the LHCII trimer in thylakoid membrane obtained from 60 µs of CG simulations show an hour-glass shape. Selected MGDG lipids are depicted whose cone shape fits well the protein shape (Thallmair et al. 2019). In **C**, an LHCII complex from plants is reported (color scheme as in Fig. 1). Solvent, membrane, and Chls’ phytol tails not shown for clarity. In Ref. (Liguori et al. 2015) a single Chl couple (Chl a611-a612) has been found to be a disordered domain (Sect. 3.2). This disorder is measured by the changes in the excitonic coupling between the two Chls, as reported in **C** for one of the simulations in Ref. (Liguori et al. 2015). Such changes depend on the different conformations and organizations experienced by Chl a611-a612 along the simulated trajectory, as shown with different colors in **C**. In **D**, the pH-dependent conformational change at Helix 3 of PsbS observed by CpHMD in Ref. (Liguori et al. 2019) is reported, as described in Sect. 3.2

Another good example is the study of the motions of quinones in and out of the reaction center. The binding affinity of electron carriers to the *Q*_A_ site was quantified in a MD work on the bacterial reaction center (Madeo et al. 2011). Via steered MD, the unbinding of quinone and of the reduced anionic semiquinone form was modeled and revealed that the two forms have a similar binding affinity, despite the slower dissociation rate of charged semiquinone (Madeo and Gunner 2005). The stability of quinones at its binding sites was also observed in the case of PQ at a CG resolution (Van Eerden et al. 2017b): PQ occupying the *Q*_A_ and *Q*_B_ sites from the start of the simulations remained stationary in both sites along the whole trajectories, as expected experimentally (Diner et al. 1988; Araga et al. 1993; Ermakova-Gerdes and Vermaas 1998). Although no spontaneous binding to the *Q*_B_ site was observed, PQ was found to enter spontaneously in PSII (Fig. 6A). Specifically, it was found that PQ takes ~ 30 μs to enter or exit from different exchange channels connected to the *Q*_B_ and *Q*_C_ sites. *Q*_C_ is an additional PQ site nearby *Q*_B_, which was recently discovered in cyanobacteria and whose role is under debate (Guskov et al. 2009). This rate is much faster than the rate of the redox steps (Kolber and Falkowski 1993; Kern and Renger 2007), ensuring a readily available pool of PQ nearby the *Q*_B_ site. Inside the exchange channels, PQ was found to be able to reorient (flip-flop), with a flip-flop time much slower (~ 100 μs) than the one observed in bulk membranes (~ 1 μs) (van Eerden et al. 2015). PQH2 reorientation was not observed in the cavities, suggesting a much slower flip-flop time for this cofactor (> 200 μs) (Van Eerden et al. 2017b). This was attributed to differences in polarity between PQ and PQH2.

The lipid-binding sites of PSII were also analyzed in detail (Van Eerden et al. 2017a). In these simulations, with individual simulation times of more than 85 μs, the immediate surrounding of PSII was found to be enriched in MGDG and SQDG. This enrichment was attributed to electrostatic interactions, as charged residues are involved in 12 of the 13 identified lipid-binding sites of PSII. Only binding sites for MGDG (nine) and SQDG (four) were found, which showed a large variety of residence times between 100 ns up to 86 µs (limited by the simulation time). Determination of the functional role of the observed lipid-PSII interactions was, however, limited by the absence of atomistic detail and, in this case, the absence of experimental comparison.

A recent coarse-grained study of plant light-harvesting complex II (LHCII) showed an enrichment of MGDG in the annular lipid shell of LHCII trimers (Thallmair et al. 2019) (Fig. 6B), similar to PSII. In contrast to PSII, the negatively charged SQDG was not enriched around the LHCII trimers, which might be due to the total charge of −18 of the antenna protein. The composition of the annular lipid shell of LHCII correlates well with the lipids associated with LHCII trimers purified in mild detergent conditions (Schaller et al. 2010). The cone shape of MGDG, which fits well the hour-glass shape of the LHCII trimer, was identified as the main reason for the MGDG preference (Thallmair et al. 2019).

### (Sub)μs timescale: fast conformational changes of the photosynthetic subunits

Conformational changes of single photosynthetic subunits can take place on the nanosecond timescale (Liguori et al. 2015, 2019; Ioannidis et al. 2016; Daskalakis and Papadatos 2017). The protein can alter its conformation by locally changing the secondary structure and/or by the motion of selected domains; the pigments and cofactors bound to the protein can also move within or away from their binding pockets and/or can undergo structural deformations. Small displacements, such as motions of protein side chains or backbone fluctuations, can also occur much faster on the ps timescale (Charlier et al. 2016).

Various MD studies have shown that there is a heterogeneity in flexibility along the structure both at the level of the photosystems and of the single LHCs: as it could be expected, the most flexible domains are the ones more exposed to the membrane/water environment, i.e., external protein subunits in the case of PSII and PSI (Ogata et al. 2013; Harris et al. 2014; van Eerden et al. 2017a, b, c) or exposed protein domains in the case of isolated LHCs (Liguori et al. 2015; Thallmair et al. 2019). The most rigid parts are instead the core protein subunits of the photosystems and the core domains of the LHCs. This heterogeneity in flexibility matches the crystallographic B factors of the structures used for the MD simulations discussed here (Jordan et al. 2001; Liu et al. 2004; Umena et al. 2011) and, also, the heterogeneity measured via NMR (Sunku et al. 2013) and electron paramagnetic resonance (EPR) spectroscopy (Dockter et al. 2012).

Importantly, structural flexibility can have a functional role in photosynthesis regulation (Ruban et al. 2012; Liguori et al. 2019) because it correlates with spectroscopic observables in several photosynthetic pigment-binding complexes (Pascal et al. 2005; van Oort et al. 2007; Liguori et al. 2013, 2017; Staleva et al. 2015; Gwizdala et al. 2016; Kondo et al. 2017). As above anticipated, single molecule (Krüger et al. 2011; Valkunas et al. 2012; Schlau-Cohen et al. 2015) and Raman spectroscopy (Pascal et al. 2005; Ruban et al. 2007) have detected conformational changes in the LHCs, but the specific domains involved in the conformational switches remained unidentified for long.

Conformational changes of an LHC were reported via MD simulations for the first time in 2015 (Liguori et al. 2015) via a set of UA simulations (GROMOS FF) on a monomeric LHCII embedded in a model lipid membrane. This work provided structural insights to a series of previous experimental findings. For example, high disorder was systematically observed via simulations at the N-terminus, in agreement with EPR/ESR results (Dockter et al. 2012; Shabestari et al. 2014). The MD simulations revealed that the motions of the N-terminus correlate with changes in the excitonic interactions of the lowest energy site of the complex, represented by a couple of Chls (Remelli et al. 1999; Novoderezhkin et al. 2005; Müh et al. 2010) (Fig. 6C). The high disorder probed at this Chl site also agrees with several experimental findings (Liu et al. 2004; Standfuss et al. 2005; Müh et al. 2010; Vrandecic et al. 2015). In the same simulations, high disorder at a Car site (neoxanthin) was observed (Liguori et al. 2015), as expected from Raman spectroscopy (Pascal et al. 2005). Finally, a loss of a hydrogen bond at a Chl b pair was observed via MD by equilibrating (and therefore solubilizing) the crystal structure in the membrane (Liguori et al. 2015). This loss matches the H-bond loss measured at a Chl b site upon solubilization of crystalline LHCII (Pascal et al. 2005). Based on these MD simulations (Liguori et al. 2015), it was then possible to propose which Chl b dimer is involved in the H-bond loss (Pascal et al. 2005).

The MD simulations also provided the possibility to study the effect of the protein environment of LHCII on the structure of the bound Cars (Liguori et al. 2017). For this work, several independent MD simulations each running for about 1 μs at UA resolution (GROMOS FF) were analyzed. It was found that Cars have a different degree of conformational freedom inside their binding pocket in LHCII and that the degree of disorder depends on the carotenoid species. This finding confirmed results from ultrafast transient absorption spectroscopy on samples of LHCII binding astaxanthin: the presence of multiple Car conformations inside LHCII was indeed detected and it was demonstrated that each Car geometry is associated with a different function in light-harvesting regulation (Liguori et al. 2017).

MD simulations at the CG resolution using the Martini force field revealed that Chls are sensitive to protein–protein interactions between their embedding LHCII complex and neighboring complexes (Thallmair et al. 2019). Chls in close proximity to protein–protein interfaces in the trimeric complex exhibit less flexibility than Chls located in proximity to the protein–membrane interface. Moreover, the simulations revealed that the average chlorophyll distance in LHCII monomers is reduced by 0.11 nm if the proteins are assembled in the trimer compared to the monomeric state (Thallmair et al. 2019). This emphasizes the importance of the trimeric state to increase the compactness of the chlorophyll packing to achieve highly efficient energy transfer.

Nowadays, different LHC genes from plants, i.e., LHCII (Liguori et al. 2015, 2017; Balevičius et al. 2017; Thallmair et al. 2019), CP29 (Ioannidis et al. 2016; Papadatos et al. 2017) and PsbS (Daskalakis and Papadatos 2017; Liguori et al. 2019), have been simulated up to the μs timescale with atomistic resolution. Most of these works have focused in particular on characterizing how the interactions among the Chls and Cars bound to LHCs change depending on the different protein conformations sampled via MD (Liguori et al. 2015; Balevičius et al. 2017; López-Tarifa et al. 2017; Maity et al. 2019). This is of particular interest because a change of interactions among Chls or between Chls and Cars can lead to creation or disruption of quenching sites inside the LHCs or can change their spectral properties. In 2017 (López-Tarifa et al. 2017), it was shown that the ideal point dipole approximation applied on MD trajectories provides a good description of the effects of protein dynamics on Chls–Chls excitonic interactions, without the need of more sophisticated QM methods. On the contrary, it was shown that QM methods are necessary to accurately describe changes in Chl-Car couplings along an MD trajectory. Ab initio protocols have been recently developed to compute Chl-Car couplings and also, more specifically, predict the activation of quenching in LHCs (Duffy et al. 2013; Balevičius et al. 2017; Fox et al. 2017; Maity et al. 2019). A brief overview of studies using MD simulations in combination with QM methods will be given in Sect. 4.2.

Another focus of the most recent MD simulations on LHCs has been to understand the effect of external factors, such as pH or the xanthophyll zeaxanthin, on the conformational dynamics of these proteins. Synthesis of zeaxanthin and acidification of the thylakoid lumen have been proposed to control the activation of photoprotective quenching in the LHCs (Demmig et al. 1987; Müller et al. 2001; Li et al. 2009). In two separate MD works, atomistic simulations (OPLS force field) of LHCII (Papadatos et al. 2017) and CP29 (Ioannidis et al. 2016) were run for hundreds of ns in which the luminal glutamic (Glu) and aspartic (Asp) acid residues were assigned a specific protonation state. The protonation pattern was chosen to model either acidic or neutral conditions in the thylakoid lumen and was kept fixed during the whole simulation. As a result of protonation of luminal residues, a conformational change was detected at the level of helix D in both LHCII and CP29 and such change was found to be able to modify the energetics of a selected Chl dimer (Ioannidis et al. 2016; Papadatos et al. 2017). Because from in vitro as well as in vivo results, no spectral differences are expected to be induced directly by pH in CP29 (Crimi et al. 2001) and LHCII (Tokutsu and Minagawa 2013; Dinc et al. 2016; Liguori et al. 2016), it could be possible that the actual pKa of CP29 and LHCII residues is too low to match the protonation pattern assigned in these simulations.

In general, protonating selected residues at the start of an MD simulation and keeping such a protonation pattern fixed throughout the simulation is a reasonable way to test how selected residues, when protonated or deprotonated, influence the dynamics of a protein. However, this choice restricts the MD sampling of the conformational space to the structures associated with that specific protonation pattern. Importantly, setting a fixed protonation pattern does not allow to simulate any specific pH values, which depend on a strict correlation between protonation states and conformations (Nielsen et al. 2011; Gunner and Baker 2016). To obtain information on the whole ensemble of protein conformations (and protonation patterns) associated with a specific pH value, non-standard MD methods need to be used. One of the most popular methods, constant pH molecular dynamics or CpHMD (Baptista and Soares 2001; Baptista et al. 2002; Machuqueiro and Baptista 2006; Oliveira et al. 2016), was applied to the stress-related complex PsbS (Liguori et al. 2019). PsbS is part of the large LHC family (Li et al. 2002a) and is pH-responsive (Li et al. 2002b, 2004), although its detailed mechanism of action is still unknown. The CpHMD work was performed at a UA resolution (GROMOS FF) by running several independent simulations at six different pH values for a total of ~ 5 μs simulated time (Liguori et al. 2019). This work showed that PsbS responds to physiologic pH changes by forming (at low pH) or losing (at high pH) a 3_10_-helix at the luminal side (helix H3), as reported in Fig. 6D. The pH-sensitive site has been proposed to be key for PsbS to form dimers with other subunits (Fan et al. 2015). Via CpHMD on PsbS (Liguori et al. 2019), it was also shown that the pH sensitivity of PsbS relies on the pKa of its Glu residues exposed to the lumen: in most cases, their pKa is strongly upshifted with respect to the standard value of Glu in water and matches the physiological pH values attained in the thylakoid lumen (Takizawa et al. 2007). Importantly, it was shown that such pKa shifts could not be obtained by continuum electrostatics calculations on the average conformation represented by the crystal structure, but only when taking into account the whole set of conformations equilibrated at each pH value via CpHMD. It is important to mention that the electrostatics of other photosynthetic subunits have been studied in great detail at a static level (without the use of MD simulations), in particular in the case of reaction centers to characterize the titrations and energetics associated to electron transfer processes, e.g., (Beroza et al. 1995; Lancaster et al. 1996; Ishikita and Knapp 2006).

Finally, the effect of pH and zeaxanthin on the dynamics of interactions between different LHC subunits was tested via standard MD. This was done in two separate works at atomistic (OPLS FF) (Daskalakis 2018) and CG (Martini FF) (Daskalakis et al. 2019) level. At atomistic level (Daskalakis 2018), by combining unbiased MD with enhanced sampling (metadynamics, see Sect. 2.2), the authors showed that PsbS can interact with CP29 in the membrane and that this interaction is more favorable at low pH. Zeaxanthin was found to occupy positions at the interface between PsbS and CP29. Possible interaction interfaces between PsbS and CP29 were also proposed. At the CG level (Daskalakis et al. 2019), it was observed that the presence of PsbS among several LHCII trimers enhances their mobility. The authors hypothesized that an enhanced mobility could possibly favor aggregation of LHCII in the thylakoid membrane, one of the mechanisms proposed to regulate activation of photoprotection (Horton et al. 2005; Ruban et al. 2012).

## Dynamics beyond the application range of MD

In this section, we discuss examples of studies that are beyond the typical application range of MD simulations (see Fig. 2). In doing so, we would like to give the reader a glimpse on some of the topics and questions which can be addressed using supra CG approaches (Sect. 4.1) and quantum mechanical methods (Sect. 4.2), respectively. In the latter case, we discuss some examples in which MD or QM/MM simulations provided protein-pigment configurations which were then used to investigate the electronic properties of the systems.

### Supra coarse-grained approaches

Even coarser representations of (bio)molecules enable the simulation of time and length scales way beyond the order of magnitude that is accessible by atomistic or CG MD simulations (see Fig. 2). This comes at the cost of molecular details and chemical specificity, which have to be sacrificed—at least partially—in the coarser representation.

In a study comparing the diffusion constant of phosphorylated and unphosphorylated LHCII trimers, each protein complex, e.g., the LHCII trimer or the photosystem II, was treated as one CG particle applying a hard-sphere model (Drepper et al. 1993). A Monte–Carlo simulation of a thylakoid membrane patch of several 100 nm was performed to compare the diffusion constant of the two types of LHCII trimers. A similar approach was applied to study the diffusion of plastoquinol in the thylakoid membrane (Tremmel et al. 2003). More sophisticated models have been developed to study the lateral organization of proteins in the thylakoid membrane (Tremmel et al. 2005; Schneider and Geissler 2013; Lee et al. 2015).

### Including electronic degrees of freedom using quantum mechanical methods

If the interest of a simulation study is not on the mesoscopic organization of the thylakoid membrane but e.g., rather on photoinduced processes and subsequent electron transfer taking place in the photosynthetic proteins, different theoretical methods are required. In this case, electronic degrees of freedom have to be considered explicitly, and thus, purely atomistic FFs are too coarse for this purpose. A common strategy is to combine a small quantum mechanically described system with a larger system treated with an atomistic FF. This method was pioneered among others by Karplus, Levitt, and Warshel who received the Nobel Prize in Chemistry in 2013 for their developments.

In many cases, atomistic MD simulations are used to sample protein dynamics on the sub-microsecond time scale around the crystal structure. Based on snapshots extracted from these simulations, quantum mechanics/molecular mechanics (QM/MM) calculations are performed to obtain detailed information about the electronic structure of the photosynthetic chromophores. Often semi-empirical QM methods are used due to the system size and the large number of calculations. In an early study, the octameric bacterial light-harvesting II (LH2) complex embedded in a model membrane was equilibrated for 2 ns before using snapshots from a picosecond-long simulation to calculate the absorption spectrum and circular dichroism spectrum of LH2 (Damjanović et al. 2002; Janosi et al. 2006; Kosztin and Schulten 2008). The simulated spectra showed that the broad absorption of the B800 ring is primarily due to fluctuations of the electric field induced by the polar environment.

Recently, a two-dimensional electronic spectrum of LH2 was presented which was modeled purely based on ab initio data (Cupellini et al. 2016; Segatta et al. 2017). The protein environment was taken into account as polarizable environment during the high-level quantum chemical calculations. In doing so it was possible to obtain an accurate description of the Car-Q_x_ spectral region of LH2. The ab initio simulated spectra reinforced the contribution of a dark state of the Cars in this energy range (Segatta et al. 2017). The study highlighted the potential of solely ab initio-based simulation protocols.

In most studies, two-dimensional electronic spectra were calculated by using experimental observables to obtain several modeling parameters as e.g., done for LH2 (van der Vegte et al. 2015) and Fenna–Matthews–Olson (FMO) complex (Olbrich et al. 2011a). Also, the dynamics in the excited states were simulated e.g., for LH2 (van der Vegte et al. 2015), LH3 (Mallus et al. 2018), and the bacterial reaction center (Vassiliev and Bruce 2006; Zhang et al. 2014; Hsieh et al. 2019) based on snapshots extracted from MD simulations. From a theoretical point of view, including the protein environment in calculations of electronic states is challenging. Example studies of its impact on the electronically excited states were performed for the FMO complex (Olbrich et al. 2011b; Vassiliev et al. 2011) and the bacterial reaction center (Narzi et al. 2016, 2017). The impact of nanosecond protein fluctuations on excitation energy transfer and charge separation were evaluated as well for bacterial reaction centers (Vassiliev and Bruce 2006; Zhang et al. 2014; Hsieh et al. 2019; Kulik et al. 2020). MD simulations coupled to quantum chemical calculations permitted also to gain insights in the couplings of selected pairs of chlorophylls (López-Tarifa et al. 2017) and excitation energy quenching via carotenoids in LHCII (Balevičius et al. 2017; Maity et al. 2019).

Besides the photoexcitation and energy transfer processes, also the different oxidation states of the oxygen-evolving cluster of photosystem II during water splitting in the Kok cycle were studied. Picosecond-long QM/MM dynamics provided new insights in the transition from the S2 to the S3 state (Bovi et al. 2013) and from the S3 to the S4 state (Narzi et al. 2018). The vibrational fingerprint of the oxygen-evolving complex was also studied using QM/MM dynamics simulations (Bovi et al. 2016) as well as the water channels surrounding the oxygen-evolving cluster (Reiss et al. 2019).

The interested reader is referred to the following reviews which provide a more detailed overview of the combination of MD simulations with quantum chemical calculations applied to photosynthetic pigment-protein complexes (Buda 2009; Neugebauer 2009; Segatta et al. 2019; Cupellini et al. 2019). For more general reviews on QM/MM methods, we refer the reader to Wanko et al. (2006) and Senn and Thiel (2009).

## Summary and outlook

With this review, we provided a basic introduction to classical atomistic and CG MD as well as an overview of their applications to photosynthetic proteins involved in the first steps of photosynthesis.

We showed that, with classical MD, it is nowadays possible to investigate the dynamics of proteins, lipids, pigments, and cofactors in the thylakoid membrane from the (sub)nanoseconds up to the ~ microseconds timescale. In particular, thanks to the development of several FFs at different levels of resolution for lipids and detergent, it is possible to study the behavior of photosynthetic protein complexes in a variety of environments that reproduce experimental conditions. In all these different conditions, we illustrated how classical MD simulations are able to give unprecedented molecular insights on the (sub)nanometer length scale in thylakoids complementing experimental findings. In doing so, MD simulations provide valuable quantitative information for the interpretation of experiments.

Importantly, the power and reliability of MD simulations very critically depend on the proper control of various conditions related to the model as well as to the simulation protocol. These conditions range from the selection of a FF compatible and validated for all components in the simulation box up to the choice of the total simulation time and the number of independent replicas which must be simulated to obtain relevant sampling.

To conclude, it must be emphasized that the large (supra)nanometer scale and (supra)millisecond timescales relevant to complex regulatory mechanisms in the thylakoid membrane represent a limit to the currently available atomistic and CG FFs. Consequently, processes such as the migration of LHCs from one photosystem to another at the base of state transitions (Allen 1995) or the full mechanisms of action of PsbS and zeaxanthin on PSII cannot (or at least have not) yet be determined. However, we can envision that future multiscale modeling, the development of increasingly more accurate CG methods, and the insertion of challenging parameters such as pH in increasingly larger and more complex simulated systems (Perilla et al. 2015; Singharoy et al. 2019) will allow MD simulations to provide structural insight on more and more complex photosynthetic processes in the thylakoid membrane.
